# Prediction of the Viscoelastic Properties of a Cetyl Pyridinium Chloride/Sodium Salicylate Micellar Solution: (II) Prediction of the Step Rate Experiments

**DOI:** 10.3390/polym14245561

**Published:** 2022-12-19

**Authors:** Shuxin Huang

**Affiliations:** 1 Department of Engineering Mechanics, Shanghai Jiao Tong University, Shanghai 200240, China; huangshuxin@sjtu.edu.cn; 2 Key Laboratory of Hydrodynamics of the Ministry of Education, Shanghai Jiao Tong University, Shanghai 200240, China

**Keywords:** micellar solution, viscoelastic property, constitutive equation, structuralized parameter, prediction

## Abstract

The reliable viscoelastic characterization and prediction of micellar solution is still required in industrial applications of the solution, e.g., in surfactant flooding and pharmaceuticals. Based on the recent theoretical characterization of the viscoelastic properties of a cetyl pyridinium chloride/sodium salicylate (CPyCl/NaSal) wormlike micellar solution with a structuralized constitutive model in the work published in 2022, the present work predicted five groups of transient shear viscoelasticities of the solution experimentally obtained in 2010, which include the first normal stress difference (N_1_) versus time curve in the start-up experiment, the shear stress (τ_12_) in the start-up experiment, τ_12_ in the long-term start-up experiment, the stress relaxation upon cessation of steady shear flow, and the transient N_1_/τ_12_ in the step strain experiment. The study findings clearly show an improvement in the predictions of the viscoelastic properties of the micellar solution compared with those predicted previously. For example, the experimental N_1_/τ_12_ is 9 at the strain of 9 in the step strain experiment, and the corresponding previous and present predictions are 2.47 and 8.45, respectively.

## 1. Introduction

Chemical flooding is an effective technology to enhance oil recovery [1,2] in the tertiary oil recovery process by increasing the viscosity of flooding fluid or reducing the interfacial tension between crude oil and reservoir brine. Surfactant flooding is a kind of chemical flooding [2,3,4] that can reduce the interfacial tension between oil and water and mobilize the trapped oil after water flooding. In recent decades, surfactant flooding has drawn increasing attention due to its merits, such as its interfacial property. Many studies have been conducted on both the physical properties of surfactant solution [1,2,3,4,5,6] and the numerical simulation of the flow of surfactant flooding [7,8]. A problem in these studies is that the present numerical simulation of surfactant flooding is not perfect, as the theoretical description of the flow property of surfactant solution still has defects. This hinders the theoretical analysis of surfactant flooding. Moreover, the theoretical viscoelastic analysis of micellar solution is also needed in other industries, e.g., cosmetic, food, and pharmaceutical.

The study status of the theoretical characterization of the viscoelastic property of micellar solution is introduced in the first part of the work of Huang [9] and indicates the deficiencies of the present theories. For example, the species-based Vasquez–Cook–McKinley model cannot describe the transient first normal stress difference of a micelle well [3], which is also shown in the present work. Also in the recent work [9], a modified Rivlin–Sawyers model was provided to characterize and predict the viscoelastic property of a micellar solution, which is based on the experimental viscoelasticity of the wormlike micellar solution, i.e., 100 mM/50 mM of CPyCl/NaSal in a 100 mM NaCl solution, as obtained by Pipe et al. [3]. The shear viscoelastic characterization of the solution is provided in full in [9], which also shows the prediction of the steady shear experiment, indicating some successful aspect of the adopted model. Due to the limitation of the length of a paper, further predictions on the viscoelastic properties of the solution are not shown in [9].

A large number of the transient viscoelastic properties of the micellar solution are shown in the experimental work of Pipe et al. [3], which provides excellent viscoelastic data for checking the theoretical model. These are predicted in the present study using the proposed model and the viscoelastic characterization of the solution in [9]. The objective of the present work is to further examine the capability of the model in [9] based on the experiment in [3].

## 2. Materials and Methods

The viscoelastic experiment of the CPyCl/NaSal solution can be found in detail in the work of Pipe et al. [3], some of which was also introduced in the recent work of Huang [9]. Five groups of the transient viscoelastic experiments of the micelle are predicted here: the N_1_ versus time curve in the start-up experiment; the shear stress τ_12_ in the start-up experiment; the shear stress in the long-term start-up experiment; the stress relaxation upon cessation of steady shear flow; and the transient N_1_/τ_12_ in the step strain experiment.

The modified Rivlin–Sawyers (RS) model [9] was used to characterize the viscoelastic property of the micellar solution, which stems from the recent works of Huang [10,11,12] and is written as:

(1) $$\tau =\int_{-\infty }^{\;t}m\left(t-t^{\prime },f,\zeta \right)\cdot h\left(\gamma \right)\cdot [\delta -C_{t}^{-1}\left(t^{\prime }\right)]dt^{\prime }$$

where *m*(*t* − *t*′, *f*, *ζ*) is the time- and shear-rate-dependent memory function with the structure effect induced by shear rate; *t* and *t*′ are the present and past time, respectively; *f* and *ζ* are two scalar structuralized parameters, respectively; *h* is a strain-dependent function; *γ* is shear strain; *δ* is an unit tensor; and ***C****_t_*^−1^ is the Finger strain tensor, i.e., the inverse of the Cauchy–Green strain tensor ***C****_t_*. The memory function *m* is written as:

(2) $$m=\sum_{i}\frac{g_{i}\cdot f(\dot{\gamma })\cdot \zeta (t,\dot{\gamma })}{\lambda_{i}}\cdot e^{\left(-\frac{t-t^{\prime }}{\lambda_{i}}\right)}$$

where *λ_i_* and *g_i_* are the relaxation times and relaxation modulus coefficients, respectively, at low shear rate or at rest; *i* is the number of relaxation spectra; and $\dot{\gamma }$ is the shear rate. The strain-dependent function *h* used is the Papanastasiou–Scriven–Macosko (PSM) function [9,13,14,15].

The model contains four groups of parameters, i.e., the relaxation spectra, the parameter in the strain-dependent function, structural parameter *f* for considering the shear-rate effect, and structural parameter *ζ* for considering both the shear-rate and shear-time effect simultaneously. All the parameters can be found in [9], where the relaxation spectra of the solution is obtained by fitting the frequency sweep experiment; the parameter in the strain-dependent function is obtained by fitting the stress relaxation experiment under step strain; parameter *f* is obtained according to the steady shear stress experiment; and parameter *ζ* is from the start-up experiment, which is calculated using the linear interpolation method. 

## 3. Results and Discussion

Based on the viscoelastic characterization of the micellar solution, the steady shear properties of the solution—such as the first normal stress difference (N_1_)—were predicted in [9], which indicates some reasonable aspect of the viscoelastic theory studied here. As mentioned in the first part of the work [9], another five groups of transient shear experiments reported by Pipe et al. [3] can also be predicted to check the capability of the model. Below are the predictions.

### 3.1. Start-Up Experiment

Figures 8, 10 and 13, reported by Pipe et al. [3], present the stress growth in the step rate experiment (i.e., the start-up experiment) of the CPyCl/NaSal solution, and the differences among the three figures can be observed in the experimental data, where the shear stress growth in Figure 8 was employed to obtain parameter *ζ*. The other three groups of experiments are predicted here.

#### 3.1.1. N_1_

**Figure 1:**
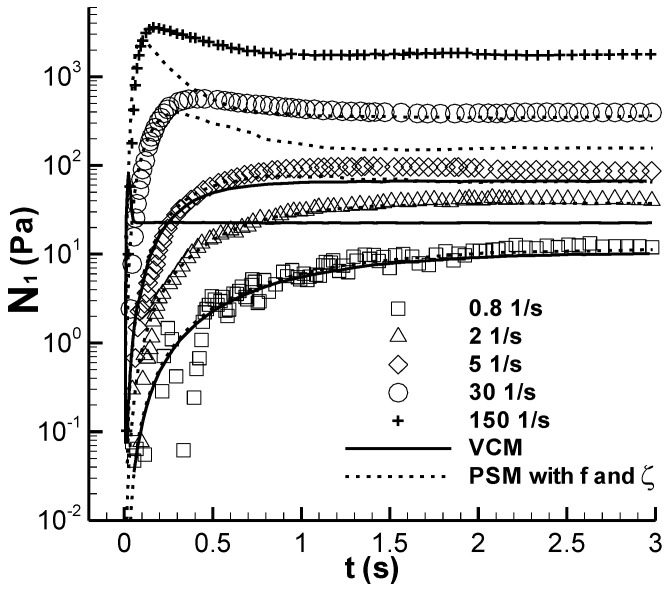
Transient N_1_ of the CPyCl/NaSal solution in the start-up experiment. Symbols are the experimental data in Figure 8 in the study conducted by Pipe et al. [3], and lines are the calculations. The calculated values for the VCM model are at 0.8, 5, and 150 s^−1^ [3], and “PSM with *f* and *ζ*” is the present calculation.

The growth of the first normal stress difference N_1_ in the step rate experiment is also shown in Figure 8, as reported in [3], which is shown here in Figure 1, along with the prediction using the Vasquez–Cook–McKinley (VCM) model and the calculated result from the modified RS-PSM model with both parameters, *f* and *ζ*. The calculated results from both models show consistency with those from the experiments performed under the shear rate of 5 s^−1^. At the shear rate of 150 s^−1^, the result of the VCM model features a small and sharp stress-overshoot regime, which exhibits a large deviation from the experiment. The results of the present calculations at 30 and 150 s^−1^ also show deviations from the experimental data, but the results are improved appearance, indicating that both the present model and the parameters describing the viscoelastic properties of the CPyCl/NaSal solution in this study can reflect the N_1_ property of the solution more effectively. When the shear time reached 2 s and the stress had a steady status, the transient N_1_ in Figure 1 was equal to that calculated in the steady shear experiment in [9]. The reason for the inefficiency of the present model in predicting N_1_ is unknown. Remarkably, Gaudino et al. [16] predicted the transient N_1_ of the same CPyCl/NaSal solution with 50 mM of NaSal at least at a steady state using the parameters obtained in characterizing the shear stress growth data. 

#### 3.1.2. Shear Stress

Figure 13, reported by Pipe et al. in [3], shows six groups of the experimental data in the step rate experiment, where three groups (the maximum shear stress in the stress growth after applying a step rate, the steady shear stress after long-term shear, and a group of stress growth data at the shear rate of 70 s^−1^) were obtained using a single step rate. The other three groups (the minimum shear stress at the second low shear rate, the steady shear stress after long-term shear, and a group of stress developing data at the shear rate of 5 s^−1^ after shearing at 150 s^−1^) were obtained using the two-step rates mode with a decreasing shear rate. Calculation of the two-step rates mode with a decreasing shear rate in this study was hindered by both the complex strain history and the deficiency of experimental data; therefore, the experiments involving the single-step rate were predicted.

**Figure 2:**
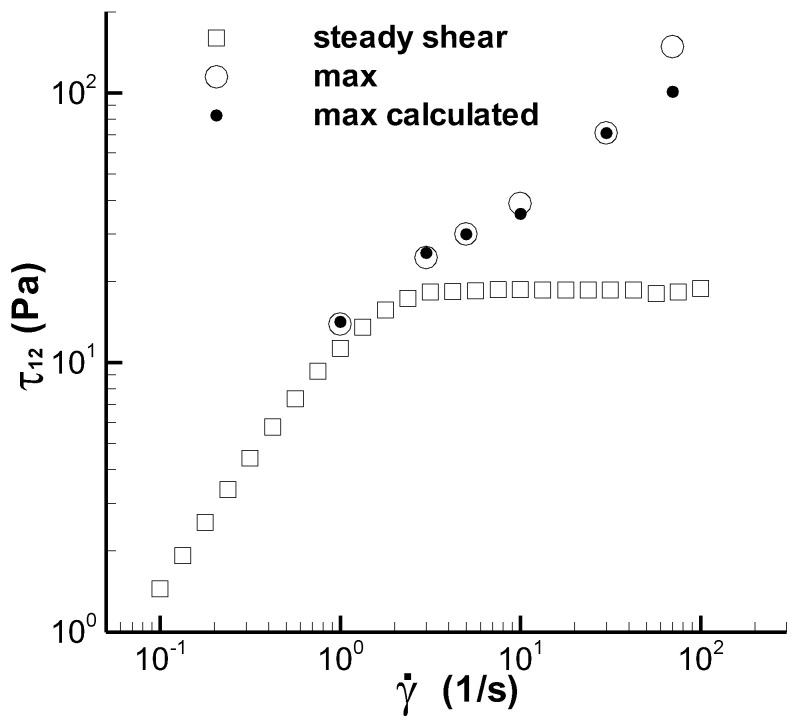
Maximum shear stress in step rate experiment and the steady shear stress of the CPyCl/NaSal solution. The square symbol is the steady experiment in Figure 6 of Pipe et al. [3], the circle is the transient experiment in Figure 13 of Pipe et al. [3], and the solid circle is the calculation here.

Figure 2 shows the maximum shear stress in the step rate, including the experimental data denoted by ‘max’ and the calculations denoted by ‘max calculated’. The values calculated at 1, 3, 10, and 70 s^−1^ are predictions, and those at 5 and 30 s^−1^ are fits. The maximum calculated value at 70 s^−1^ was approximately 33% lower than that obtained experimentally, which is attributed to the linear interpolation between the *ζ* values at 30 and 150 s^−1^ and the large gap between those at the two shear rates.

**Figure 3:**
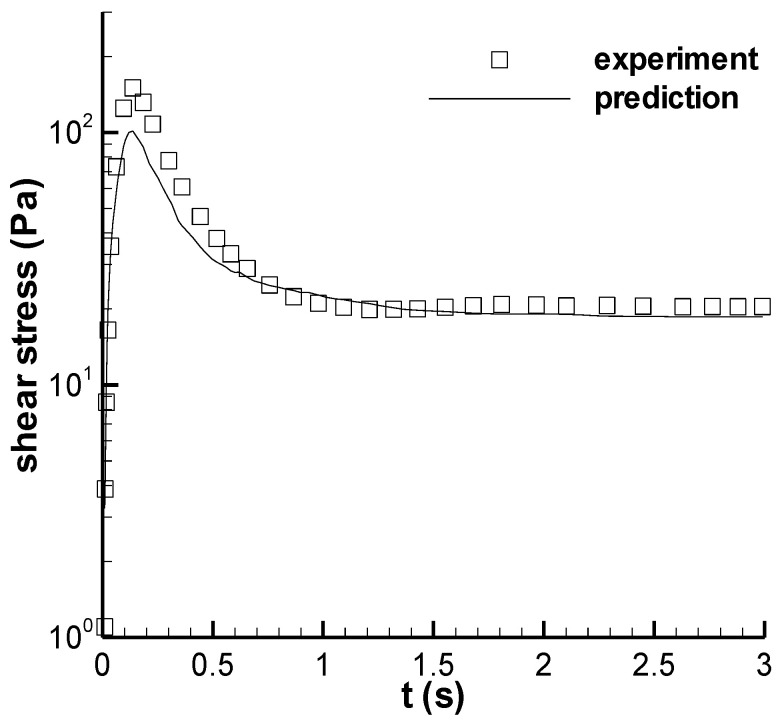
Prediction of the stress growth at 70 s^−1^. The symbol is the experiment in Figure 13 of Pipe et al. [3], and the line is the calculation here.

The prediction of the shear stress growth at the shear rate of 70 s^−1^ is shown in Figure 3. In the stress-overshoot regime, the calculation result is lower than the experimental result due to interpolation; however, in a different regime, the calculated result is consistent with the experiment result. From the *ζ* curve in Figure 5 in the first part of the work [9], we can see that the *ζ* values at both 30 and 150 s^−1^ in the overshoot regime are apparently larger than 1, which can cause corresponding calculation deviation at 70 s^−1^, and those in the steady shear regime approach 1, which will produce a calculation result similar to the experiment.

#### 3.1.3. Long-Term Start-Up Experiment

**Figure 4:**
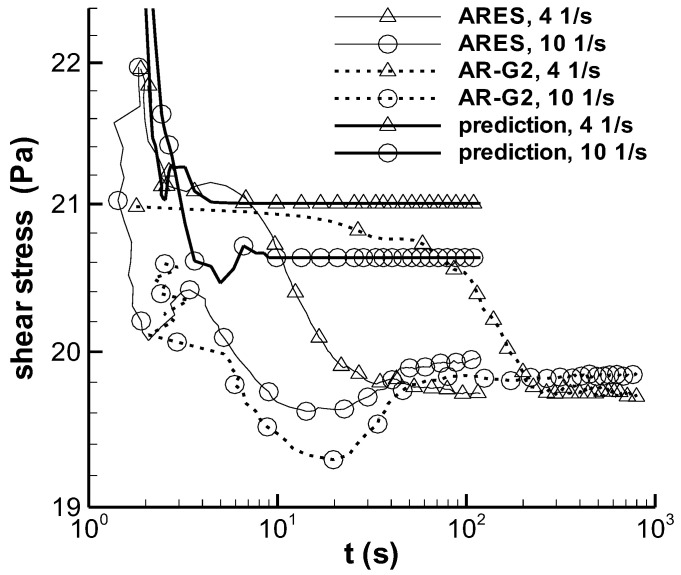
Long-term stress growth. Both the solid and the dashed lines are the experiments in Figure 10 of Pipe et al. [3], and the bold solid line is the calculation here.

Figure 10 in the study by Pipe et al. [3] presents a group of the experiments performed to show the difference between the step rate experiments obtained on two apparatuses, i.e., ARES and AR-G2 rheometer, and does not show the calculation of the VCM model. In this experiment group, the shearing time was long: 120 s for ARES and 800 s for AR-G2, and the shear rates were 3, 4, 5, and 10 s^−1^. The present modified RS-PSM model with parameters *f* and *ζ* was employed to calculate the two experiments at 4 and 10 s^−1^, shown in Figure 4. Under long-term shearing, the experimental stress exhibited a slightly decreasing trend and other subtle phenomena, and the calculated stress at 4 s^−1^ was 21 ± 0.5 Pa after the shear time reached 2 s. For the calculation at 10 s^−1^, reaching the steady state took slightly longer (approximately 2.5 s), and the stress was 21 ± 0.5 Pa. The calculated stress was constant at approximately 10 s and did not exhibit subtle variation after 10 s, despite the slight difference between the steady stresses at the two shear rates. The deviation between the calculated and experimental results was small, e.g., the deviation between the calculated steady stress and the minimum experimental shear stress was less than 7%. The maximum deviation in this case was approximately one-fifth of the deviation between the calculation result and the experiment result of the maximum overshoot stress in Figure 3.

### 3.2. Stress Relaxation Experiment

**Figure 5:**
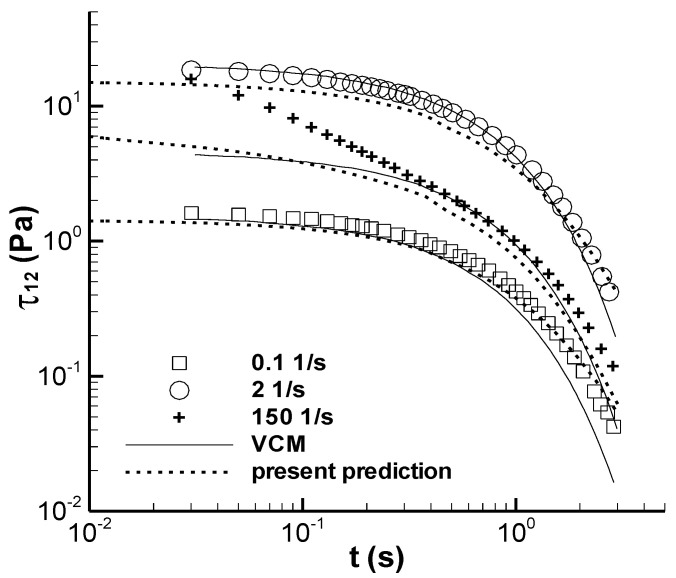
Stress relaxation property of the CPyCl/NaSal solution after steady shear. The symbols represent the experimental data in Figure 9 reported by Pipe et al. [3], and lines represent the calculated results. The solid line is the prediction of Pipe et al. [3], and the dashed line is the present calculation.

The stress relaxation experiment was conducted by first applying a step rate. Subsequently, the shear rate lasted a while until it reached a steady state for shear stress. Finally, the shearing was completed, but the recording of the shear stress variation over time was maintained. The stress in the sample upon cessation of the shearing decreased over time, and this phenomenon is called stress relaxation after steady shear. Pipe et al. [3] presented five groups of the stress relaxation experiments and three calculations of the VCM model at 0.1, 2, and 150 s^−1^. The present study also presents three calculations in the same conditions as those used by Pipe et al. [3], which are shown in Figure 5. The predictions of the present model are similar to those of the VCM model at the three shear rates; however, certain deviations can be observed between the experimental and calculated results. Pipe et al. [3] also used a two-mode exponential relaxation process to adequately describe the relaxation experiment at 150 s^−1^; however, the structuralized model in the present study was not modified using this method to improve the calculation results.

### 3.3. Step Strain Experiment

In the study by Pipe et al. [3], Figure 5 shows two groups of experimental data, i.e., the relation between N_1_/τ_12_ and time t and the relation between N_1_/τ_12_ and strain γ in the step strain experiment, as well as the prediction of the VCM model. According to the calculation process of the VCM model [3], the strain history of the step strain experiment can be formed by applying a triangular-like shear rate, such that the strain increases and reaches a constant at the end of shearing. Therefore, the strain application process in the calculation is indeed an applying-rate process, where the shear rate is applied according to a designed rule. The complex application of the shear rate used in the study by Pipe et al. [3] was not possible in this study, and the step rate mode was used to generate a strain on the sample. This is why the step rate was added to the experimental mode of this group of experiment in Table 1 [9].

Three modes of shear rate application were employed here to generate the seven strains used in the step strain experiment conducted by Pipe et al. [3]. The first application of the shear rate of 150 s^−1^ was denoted by “Mode 1”, and the shear time used was calculated using the given strain. The shear time t_0_ in Table 1 is obviously long as the strain is large. In terms of the N_1_/τ_12_ versus t experiment in Figure 5 reported by Pipe et al. [3], the shear stress approached a steady state or the maximum level at approximately 0.1 s. Therefore, the second mode, denoted by “Mode 2”, involves the use of 0.1 s as the shear time. Therefore, the shear rate can be calculated by dividing the strain by the shear time. Finally, the shear time of 0.1 s is adjusted manually and slightly according to the calculations of Mode 2 and the N_1_/τ_12_ experiments, which is denoted by “Mode 3” in Table 1.

**Figure 6:**
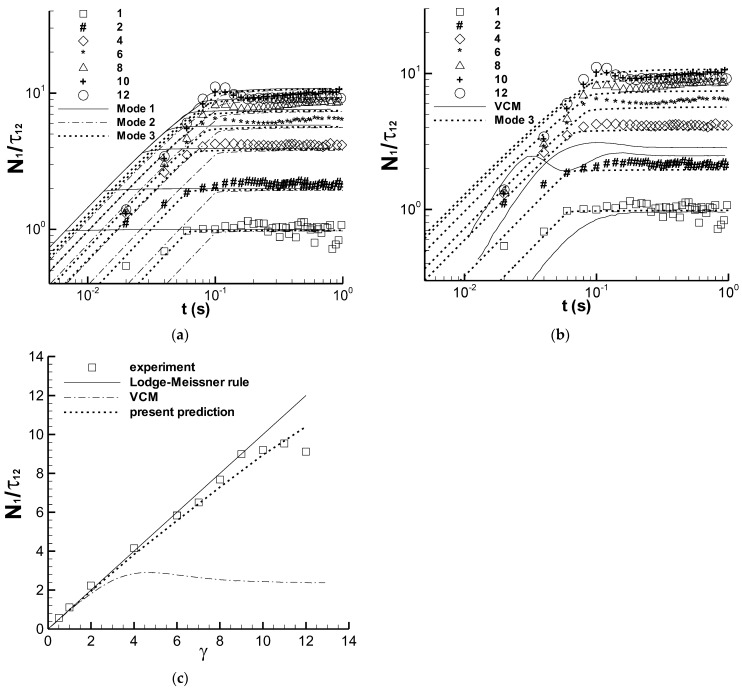
(**a**) Relation of N_1_/τ_12_ versus t obtained using three shear rate modes, (**b**) N_1_/τ_12_ of both Mode 3 and VCM model, (**c**) the correlation between N_1_/τ_12_ and γ. The symbols represent the experimental data in Figure 5 of Pipe et al. [3], and the lines are calculations, in which “VCM” and “Lodge-Meissner rule” are from Pipe et al. [3], and “Mode 1”, “Mode 2”, “Mode 3”, and “present prediction” are the present calculations.

Figure 6 shows the calculated N_1_/τ_12_ versus t relation in the CPyCl/NaSal solution using the three shear rate modes in Table 1 and the experimental and calculated N_1_/τ_12_ versus γ relation. The steady N_1_/τ_12_ values of the three shear rate modes are almost identical and almost correspond to the experimental data in Figure 6a. The difference between the calculations of the three modes is the time taken to reach a steady state of N_1_/τ_12_, which is equal to the shear time t_0_. The overshoot of N_1_/τ_12_ is also not observed in the calculation, and the N_1_/τ_12_ calculated is almost constant after t_0_. However, the experimental N_1_/τ_12_ exhibits an overshoot phenomenon at large strain and approaches a constant after 0.1 s, but it is not stable. Figure 6b shows the results of the VCM model, where the curve of the VCM model at γ = 1 is consistent with the experiment after 0.1 s, and the other two curves at γ = 6 and γ = 12, respectively, show large deviations from the corresponding experiments. The results of Mode 3 are consistent with those of the experiments, including both the steady N_1_/τ_12_ value and the time reaching the steady state or the maximum stress ratio. The consistency of time between the experiment and the calculation is attributed to the artificial adjustment of shear time according to the experiment; however, this consistency also reflects the capability of the present modified RS-PSM model with parameters *f* and *ζ* to fairly express the viscoelastic property of the micellar solution.

Figure 6c shows a clear deviation between the results of the experiment and the calculation of the VCM model, and the experimental data are consistent with the Lodge–Meissner relation, i.e., N_1_/τ_12_ = γ, under the strain of 9, which indicates some deficiency of the VCM model. The present calculation using Mode 3 approaches both the experiment and the Lodge–Meissner relation, which shows a certain reasonable aspect of the present model. In the analysis of the data on N_1_/τ_12_ versus γ, Pipe et al. [3] proposed that the deviation between the calculation of the VCM model and the experimental result could be related to the inhomogeneous flow of the solution, and the present calculation shows another possible explanation of such a viscoelastic phenomenon, i.e., the homogeneous flow could produce more of the experimental phenomena of N_1_/τ_12_ versus γ curve owing to the viscoelastic property of the solution. In addition, the influence of applying the real shear strain in the calculation remains unknown, despite the attempted application of three modes, because all three modes differ from the experimental process used by Pipe et al. [3]. This could complicate the present calculation.

Theoretical analysis on the viscoelastic property of micellar solution is an attractive topic, and at least seven such papers have been composed in 2022 [9,17,18,19,20,21,22]. The work of Pipe et al. [3] was cited in refs. [9,17,19,20,21], in which ref. [9]—i.e., the first part of the present work—showed a detailed characterization on the viscoelasticity of the micellar solution of the authors, albeit using a different method. The authors of the other four papers [19,19,20,21] only introduced the work of Pipe et al. [3] without analyzing the experimental data, using the viscoelastic theoretical model. Therefore, the present predictions on the viscoelastic properties of the micellar solution of Pipe et al. can promote the understanding on the abundant experimental viscoelastic behaviors of the micellar solution; moreover, they can further show the capability of the adopted model, although the viscoelastic behaviors of the solution still cannot be fully described by it.

## 4. Conclusions

The theoretical characterization on the viscoelastic properties of the CPyCl/NaSal wormlike micellar solution at 22 °C was employed in this study to predict five groups of transient shear viscoelastic behaviors of the solution, which are summarized here.

(1) The prediction of the transient N_1_ in the stress growth experiment was improved.

(2) The structuralized model in this study yielded reasonable results with respect to the maximum shear stress in the start-up experiment. The stress growth calculated at 70 s^−1^ was low for the maximum shear stress in the stress-overshoot regime owing to the deficiencies of both *ζ* data and the linear interpolation method. During the long-term shearing in the start-up experiment, the model yielded a steady state value, and the experiment showed a slight decrease or variation, but the deviation between the steady calculated stress and the minimum experimental stress was less than 7%.

(3) The present calculation results are similar to those of the VCM model on the stress relaxation upon cessation of steady shear flow.

(4) Both the experiment of N_1_/τ_12_ versus γ and the Lodge–Meissner relation under the strain of 9 in step strain can be adequately expressed by the model.

In summary, the prediction capability of the present model with respect to the stress relaxation experiment approached that of the VCM model, and the other four groups of predictions were clearly improved (see Figure 1 and Figure 6) or firstly shown (see Figure 2, Figure 3 and Figure 4). For example, the experimental N_1_/τ_12_ was 9 at the strain of 9 in the step strain experiment in Figure 6c, and the corresponding previous and present predictions were 2.47 and 8.45, respectively. These results indicate that the model and parameters in the present study are relatively more suitable for describing the viscoelastic properties of the CPyCl/NaSal solution at 22 °C.

In Figure 7 of the paper published by Pipe et al. [3], the second normal stress difference coefficient, which was neither used nor predicted in the present study, is shown. The method for including the effect of the second normal stress difference N_2_ in the RS equation [14,23] was not included in the present model; therefore, the N_2_ experiment was not used. Moreover, another group of data—i.e., Figures 11 and 12 reported by Pipe et al. [3]—that were obtained by applying designed stress were not predicted. In the present model, stress is a function of deformation or shear rate; therefore, the model cannot be used to calculate the shear rate by inputting the shear stress. The step stress experiment should be calculated in future studies.

## Figures and Tables

**Table 1:** Three modes of applying the shear rate.

γ	Mode 1		Mode 2		Mode 3	
	$\dot{\gamma }$ (s^−1^)	t_0_ (s)	$\dot{\gamma }$ (s^−1^)	t_0_ (s)	$\dot{\gamma }$ (s^−1^)	t_0_ (s)
1		0.006667	10		16.67	0.06
2		0.01333	20		33.33	0.06
4		0.02667	40		61.54	0.065
6	150	0.04000	60	0.1	75	0.08
8		0.05333	80		100	0.08
10		0.06667	100		125	0.08
12		0.08000	120		133.3	0.09

## Data Availability

The data that support the findings of this study are available within the article.
